# Supplementary material for: Environmental Exposure to Bisphenol A Enhances Invasiveness in Papillary Thyroid Cancer
Source: Int J Mol Sci. 2025 Jan 19;26(2):814. doi: 10.3390/ijms26020814 (PMC11766120; doi:10.3390/ijms26020814)
Supplement: Supplementary file 1 [file ijms-26-00814-s001.zip › ijms-3351258-supplementary.pdf]

Supplementary Information

Environmental Exposure to Bisphenol A Enhances Invasiveness in Papillary Thyroid Cancer

Chien-Yu Huang [1,2,†], Ren-Hao Xie [1,2,†], Pin- Hsuan Li [2,†], Chong-You Chen [1,2], Bo-Hong You [2], Yuan-Chin Sun [2], Chen-Kai Chou [3,4], Yen-Hsiang Chang [4, 5], Wei-Che Lin[6,*] and Guan-Yu Chen[1,2,7,8,*]

1 Department of Electrical and Computer Engineering, College of Electrical and Computer Engineering, National Yang Ming Chiao Tung University, Hsinchu 300, Taiwan; apple21038526.ee08@nycu.edu.tw (C.-Y.H.)
2 Institute of Biomedical Engineering, College of Electrical and Computer Engineering, National Yang Ming Chiao Tung University, Hsinchu 300, Taiwan
3 Division of Endocrinology and Metabolism, Department of Internal Medicine, Kaohsiung Chang Gung Memorial Hospital, Kaohsiung, 833, Taiwan
4 Chang Gung University College of Medicine, Kaohsiung, 833, Taiwan
5 Department of Nuclear Medicine, Kaohsiung Chang Gung Memorial Hospital, Kaohsiung, 833, Taiwan
6 Division of Neuroradiology, Department of Diagnostic Radiology, Kaohsiung Chang Gung Memorial Hospital, Kaohsiung, 833, Taiwan
7 Center for Intelligent Drug Systems and Smart Bio-devices (IDS^{2}B), National Yang Ming Chiao Tung University, Hsinchu, 300093, Taiwan
8 Department of Biological Science and Technology, National Yang Ming Chiao Tung University, Hsinchu 300, Taiwan
† These authors contributed equally to this work.
* Correspondence: guanyu@nycu.edu.tw (G.-Y.C.); Tel.: +886-3-573-1920
.

Contents

Figure S1. MDA-T32 spheroids formed with cell densities of 200 and 1000 cells/well on days 1 and 4.
Figure S2. Invasion pattern of TPC-1 and BCPAP spheroid.

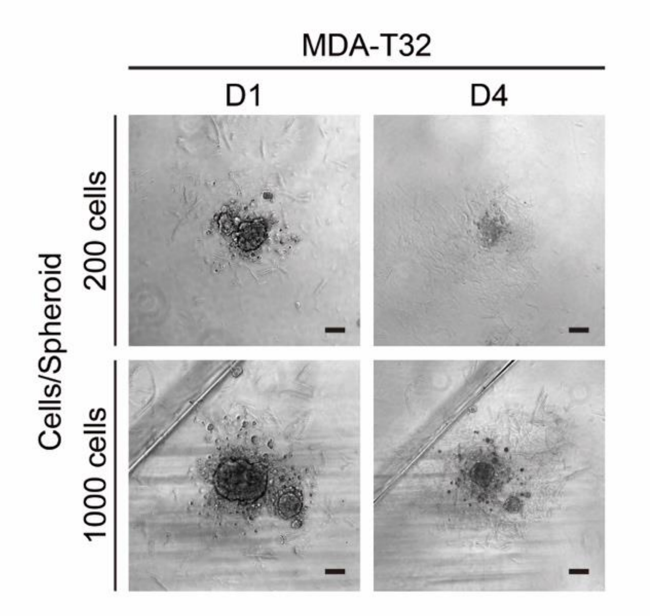

Figure S1. Quantification of Small Airway Epithelial Differentiation. The quantitative of ciliated and goblet cells expression. Scale bar: 100 μm.

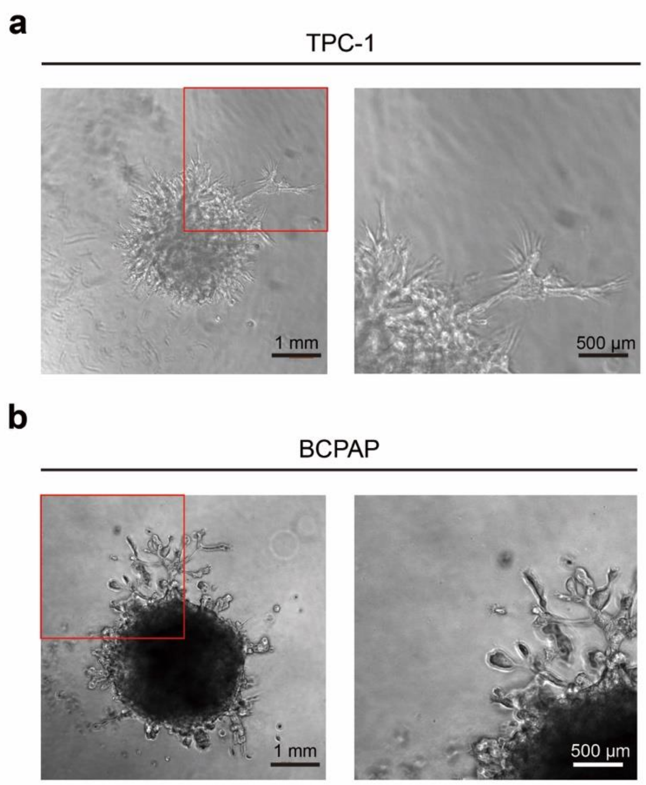

Figure S2. Invasion pattern of TPC-1 and BCPAP spheroid. (a) Bright-field images of invasion protrusion of TPC-1 spheroid. (b) Bright-field images of invasion protrusion of BCPAP spheroid.
